# Multivariate Analysis on Seven-Year Effects of Balanced N-P-K-Mg Fertilization on Productivity and Leaf Spot Incidence in Two Sweet Cherry Cultivars

**DOI:** 10.3390/plants15101499

**Published:** 2026-05-14

**Authors:** Ádám Csihon, Imre J. Holb

**Affiliations:** 1 Institute of Horticulture, Faculty of Agricultural and Food Sciences and Environmental Management, University of Debrecen, Böszörményi Str. 138, 4032 Debrecen, Hungary; csihonadam@agr.unideb.hu; 2 HUN-REN, Centre for Agricultural Research, Plant Protection Institute, Herman Ottó út 15, 1022 Budapest, Hungary

**Keywords:** nutrient, tree and yield parameters, linear regression, principal component analysis

## Abstract

Long-term balanced mineral fertilization is essential for sustainable sweet cherry production under variable climatic conditions. This seven-year field study (2016–2022) evaluated the effects of NP, NPK, and NPKMg fertilization including the control on six parameters: trunk cross-sectional area (TCSA), fruit yield (FY), crop load (CL), fruit diameter (FD), water-soluble dry matter content (BRIX), and cherry leaf spot incidence (CLS) in two sweet cherry cultivars (‘Vera’ and ‘Carmen’). TCSA increased continuously in both cultivars, while fertilization effects on growth, FY, CL, and FD varied among years and were significantly higher under NPK and NPKMg treatments compared with the control, particularly in specific years. Leaf spot incidence was reduced in the NPKMg treatment in epidemic years, although strong interannual and cultivar-dependent variability was observed, with ‘Carmen’ being more susceptible than ‘Vera’. Correlation and regression analyses revealed significant relationships among key traits, particularly for CL vs. FY, FD vs. CLS, TCSA vs. CLS, and BRIX vs. CL, indicating strong vegetative–generative interactions. Principal component analyses further showed that tree and fruit traits as well as disease incidence were structured along a limited number of integrated multivariate components explaining most of the variance. In conclusion, balanced fertilization improved productivity and partly reduced disease incidence, but treatment effects were strongly influenced by complex multivariate interactions and interannual climatic variability. These findings highlight the importance of integrative analytical approaches to optimize nutrient management under Central European conditions.

## 1. Introduction

Sweet cherry (*Prunus avium* L.) is a major temperate fruit crop with high economic and nutritional value, widely cultivated across regions characterized by moderate climatic conditions. Commercial production is primarily concentrated between 35° and 55° latitude, where winter chilling requirements and favorable spring–summer conditions support optimal flowering, fruit set, and fruit development [1]. Over recent decades, sweet cherry production has expanded considerably, both in traditional growing regions and in newly established orchards that adapt to changing climatic conditions and market demands [2,3,4]. According to FAO statistics, global cherry production reached approximately 2.9 million tons, reflecting a continuous upward trend driven by increasing consumer demand for high-quality fresh fruit [5].

The increasing adoption of intensive orchard systems has also led to greater sensitivity of sweet cherry trees to environmental constraints, including nutrient availability, water supply, and extreme weather events. These factors interact strongly with orchard management practices, such as irrigation, pruning, crop load regulation, and fertilization, ultimately determining productivity and fruit quality [6,7,8,9,10]. In intensive sweet cherry tree systems, nutrient management must reconcile productivity with environmental concerns, particularly regarding nitrogen losses, soil fertility, and resource-use efficiency [11]. In sweet cherry, the importance of a balanced nutrient supply is further emphasized by cultivar-specific characteristics of growth and fruiting. Nutrient uptake and distribution are highly dynamic processes, influenced by phenological stage, rootstock–scion interactions, soil properties, and orchard management practices [12,13].

Mineral fertilization remains one of the primary tools for ensuring adequate nutrient availability. Macronutrients such as nitrogen (N), phosphorus (P), potassium (K), calcium (Ca), and magnesium (Mg) support vegetative growth and reproductive development, regulating key physiological processes such as photosynthesis, carbohydrate partitioning, enzyme activity, and cellular structure formation, thereby directly influencing tree vigor, yield, and fruit quality [14,15].

Nitrogen is a primary driver of vegetative growth, influencing cell division, expansion, and photosynthetic capacity [11,16]. In sweet cherry, N fertilization enhances shoot growth, leaf area, and canopy density, increasing photosynthetic surface and leaf chlorophyll content [17,18,19]. However, excessive N supply often leads to vigorous vegetative growth at the expense of reproductive development, altering assimilate partitioning and potentially reducing fruit quality [20,21]. Denser canopies induced by high N inputs reduce light penetration and modify microclimatic conditions, affecting flower bud initiation, fruit set, and fruit development [22,23]. Phosphorus is critical for energy metabolism, root development, and reproductive processes [12,24]. It promotes root growth, branching, nutrient and water uptake, and supports flower bud initiation and fruit set [4]. Potassium regulates osmotic balance, enzyme activation, and carbohydrate transport, influencing fruit size, weight, sugar accumulation, firmness, and color development [13,14,25,26]. It also contributes to stomatal function and water relations under variable environmental conditions [10]. Magnesium, as the central atom of chlorophyll, is indispensable for photosynthesis and carbon metabolism. It activates enzymes associated with carbohydrate metabolism and phloem loading, facilitating assimilate transport to growing tissues and fruits [11,26].

The balance among N, P, K, and Mg is critical for physiological equilibrium, as imbalances can reduce nutrient use efficiency and tree performance [14,27]. Cultivar-specific differences further modulate responses to fertilization [15,20,27,28]. In intensive sweet cherry production, achieving an optimal vegetative–generative balance, influenced by nutrient supply and crop load, is central to productivity and fruit quality [6,9,20,26,29]. Early fruit development involves a short cell division phase (~2 weeks) followed by rapid expansion, during which fruits act as strong sinks for carbohydrates; thus, balanced nutrient supply significantly contributes to fruit size, sugar accumulation, and overall quality [6,9,20,21,26,29]. Fruit quality, encompassing size, firmness, color, soluble solids, and flavor, is strongly influenced by nutrient availability, water supply, and canopy structure [4,25,30,31,32].

In addition to growth and quality, nutrient supply also affects tree susceptibility to pests and diseases as nutrient imbalances or deficiencies reduce photosynthesis and increase susceptibility [33,34]. Potassium and calcium strengthen cell walls and improve disease resistance [35]. Excess nitrogen often increases fungal susceptibility [35] and enhances leaf spot disease [34]; vigorous shoot growth elevates leaf susceptibility [36].

Despite extensive studies on the effects of nutrient supply on cherry vegetative and generative parameters, long-term, multiyear investigations integrating tree growth dynamics, fruit quality, and leaf spot incidence remain limited. We hypothesize that balanced NPKMg fertilization enhances sweet cherry vegetative–generative performance, improves fruit quality, and reduces leaf spot incidence. Accordingly, this seven-year study aimed to evaluate the effects of four fertilizer treatments (NP, NPK, NPKMg, and control) on five tree parameters (trunk cross-sectional area, fruit yield, crop load, fruit diameter, and water-soluble dry matter content), as well as cherry leaf spot incidence, and to explore multivariate relationships among these parameters to provide predictive insights for optimized production in two sweet cherry cultivars, ‘Vera’ and ‘Carmen’. Emphasis is placed on disentangling cultivar-specific responses and integrated trait interactions to better understand the nutrient-driven regulation of orchard performance.

## 2. Results

### 2.1. Analysis of Variance

Analysis of variance (ANOVA) revealed that trunk cross-sectional area (TCSA), fruit yield (FY), crop load (CL), fruit diameter (FD), water-soluble solid content (BRIX), and cherry leaf spot incidence (CLS) were all significantly affected (*p* < 0.05) by fertilizer treatment, cultivar, and year (Table 1).

Significant interaction effects were detected between cultivar and year (C × Y) for TCSA, and between cultivar and fertilizer treatment (C × F) for FY, CL, and FD. In contrast, Brix values were significantly influenced by all interaction terms (C × F, C × Y, F × Y, and C × F × Y). No significant interaction effects were observed for CLS (Table 1).

### 2.2. Main Meteorological Parameters

During the study period from January 2016 to December 2022, the monthly mean temperature ranged from −6.0 to 25 °C (Table S1), indicating considerable interannual and seasonal variability in thermal conditions. The lowest minimum temperatures revealed the occurrence of late spring frosts in April (below −2 °C) in 2020, 2021, and 2022, which may have posed a risk to flowering and early fruit development. Total annual precipitation varied substantially across years, ranging from 397 to 720 mm (Table S1), reflecting differences in water availability during the growing seasons.

### 2.3. Trunk Cross-Sectional Area

Over the 7-year study period, TCSA increased steadily in both cultivars, with consistently higher mean values in cv. ‘Vera’ than in cv. ‘Carmen’ (Table 2).

In cv. ‘Vera’, TCSA increased from 68.0 to 119.7 cm^2^ in 2016 to 148.5–342.8 cm^2^ in 2022, while in cv. ‘Carmen’ it rose from 77.0 to 89.0 cm^2^ to 183.7–265.5 cm^2^ over the same period (Table 2).

Significant differences among fertilizer treatments were observed for both cultivars in all years (Table 2). In cv. ‘Vera’, NP and NPK treatments consistently produced significantly higher TCSA values than the Control, while NPKMg exceeded the Control from 2017 onward. In cv. ‘Carmen’, NP and NPK were significantly higher than the Control in most years (except 2016 and 2020), whereas NPKMg did not differ significantly from the Control. Across cultivars (‘overall cultivars’), NP and NPK consistently showed higher TCSA values than the Control throughout the study period, while NPKMg differed significantly from the Control only in 2018 and from 2020 to 2022 (Table 2).

In terms of annual TCSA increments (Table 3), trunk cross-sectional area increased continuously in both cultivars. In cv. ‘Vera’, annual increments ranged from 10.8 to 45.2 cm^2^, while in cv. ‘Carmen’ they ranged from 19.6 to 36.2 cm^2^ (Table 3).

The lowest annual increment was 9.2 cm^2^ in 2018 for cv. ‘Vera’ and 6.2 cm^2^ in 2018 for cv. ‘Carmen’, while the highest values were 45.2 cm^2^ in 2017 for cv. ‘Vera’ and 36.2 cm^2^ in 2019 for cv. ‘Carmen’ (Table 3). Across all treatments (‘overall treatments’), annual increments were consistently higher in cv. ‘Vera’ than in cv. ‘Carmen’ throughout the study period (Table 3).

Regarding treatment effects on annual increments, cv. ‘Vera’ showed consistently higher increases under NPK and NPKMg treatments compared with the Control. In cv. ‘Carmen’, treatment effects on annual increments were less consistent across years. Across cultivars (‘overall cultivars’), NPK treatment generally resulted in the highest annual TCSA increments compared with the Control (Table 3).

### 2.4. Fruit Yield per Tree

During the 7-year period, the highest fruit yield was recorded in the NP treatment in 2022 for cv. ‘Carmen’ (38.4 kg tree^−1^, corresponding to 30.72 t ha^−1^), while the lowest was observed in the Control treatment in 2022 for cv. ‘Carmen’ (0.5 kg tree^−1^, corresponding to 0.4 t ha^−1^) (Table 4). The yield variability of cvs. ‘Vera’ and ‘Carmen’ ranged between 1.5 and 28.7 and between 0.5 and 38.4 kg tree^−1^, respectively. In the ‘overall (treatments)’, fruit yield differed significantly between the two cultivars in each year (*p* = 0.05); however, the cultivar with the significantly higher yield varied by year, alternating between ‘Vera’ and ‘Carmen’ (Table 4).

Significant differences in fruit yield values among the four fertilizer treatments were found for both cultivars in all years, with the exception of 2022 and 2017 for cv. ‘Vera’ and ‘Carmen’, respectively (Table 4). The yield values of cv. ‘Vera’ in the NP, NPK, and NPKMg treatments were significantly higher than those of the control treatment in 2016, 2017, 2018, and 2019, while for cv. ‘Carmen’, this was observed in 2018, 2019, 2021, and 2022 (Table 4).

The overall fruit yield values for the four fertilizer treatments (‘overall cultivars’) showed that NP, NPK, and NPKMg treatments were significantly higher compared to the control treatment, with the exception of 2020 and 2021 (Table 4).

### 2.5. Crop Load

Similarly to fruit yield, both cultivars exhibited a wide range of crop load values, ranging from 9.5 to 292.5 g cm^−2^ for cv. ‘Vera’ and from 3.4 to 207.9 g cm^−2^ for cv. ‘Carmen’ over the 7-year period (Table 5). The highest crop load was recorded in 2016 in the NPKMg treatment for both cultivars (292.5 g cm^−2^ for cv. ‘Vera’), while the lowest value was observed in the control treatment in 2020 for cv. ‘Carmen’ (3.4 g cm^−2^). The ‘overall (treatments)’ data showed that the crop load of the two cultivars was significantly different in all years, with the exception of 2017 and 2019 (*p* = 0.05) (Table 5).

The values of the NPKMg treatment were significantly higher than those of the control treatment for cv. ‘Vera’ in 2016–2019 and for cv. ‘Carmen’ in 2016, 2017, 2019, 2020, and 2022 (Table 5).

The overall values of crop load for the four fertilizer treatments (‘overall cultivars’) showed no significant differences in 2018 and 2022. In 2016, 2017, and 2019, the values of the NP, NPK, and NPKMg treatments were significantly higher compared to those of the control treatment, while in 2020 and 2021 the control treatment was not significantly different from the three fertilizer treatments (Table 5).

### 2.6. Fruit Diameter

During the assessed period, the fruit diameter of cv. ‘Vera’ remained below the required 28 mm, whereas cv. ‘Carmen’ reached the optimal fruit diameter each year, except in 2022 (Table 6). Over the 7-year period, fruit diameter ranged from 21.3 to 27.5 mm for cv. ‘Vera’ and from 25.7 to 36.0 mm for cv. ‘Carmen’. The smallest fruit (21.3 mm) was harvested in 2022 from the Control treatment for cv. ‘Vera’, while the largest fruit (36.0 mm) was collected in 2020 from the NPKMg treatment for cv. ‘Carmen’ (Table 6).

The ‘overall (treatments)’ data showed that fruit diameter was significantly higher in cv. ‘Carmen’ than in cv. ‘Vera’ in all years (Table 6).

Significant differences in fruit diameter among the four fertilizer treatments were observed for cv. ‘Vera’ during 2018–2022, and for cv. ‘Carmen’ during 2016–2018 and in 2021 (Table 6). For cv. ‘Vera’, significantly higher fruit diameters were recorded in the NP, NPK, and NPKMg treatments compared with the Control between 2020 and 2022. In 2018, fruit diameter was significantly higher in the NP and NPK treatments, and in 2019 for the NPK treatment, compared with the untreated trees. For cv. ‘Carmen’, fruit diameter in the NPKMg treatment was significantly higher than in the Control in 2016, 2017, and 2019, but not in 2021 (Table 6).

The overall values for the four fertilizer treatments (‘overall cultivars’) showed no significant differences among the treatments in any year (Table 6).

### 2.7. Water-Soluble Dry Matter Content

Water-soluble dry matter content ranged from 13.58 to 22.30 Brix% for cv. ‘Vera’ and from 12.80 to 20.26 Brix% for cv. ‘Carmen’ (Table 7).

The lowest value (12.80 Brix%) was measured in the NPKMg treatment of cv. ‘Carmen’ in 2016, whereas the highest value (22.30 Brix%) was observed in the Control trees of cv. ‘Vera’ in 2019. In the ‘overall (treatments)’, water-soluble dry matter content differed significantly between the two cultivars from 2019 to 2022 (*p* = 0.05); however, the cultivar with the significantly higher dry matter content varied by year, alternating between cvs. ‘Vera’ and ‘Carmen’ (Table 7).

For cv. ‘Vera’, water-soluble dry matter content in the NP, NPK, and NPKMg treatments was significantly lower than in the Control treatment in all years, with the exception of 2018. For cv. ‘Carmen’, this pattern was observed in 2016, 2017, 2020, 2021, and 2022, whereas in 2018 and 2019, the NPKMg treatment had significantly higher values than the Control (Table 7).

The overall water-soluble dry matter content values for the four fertilizer treatments (‘overall cultivars’) showed that the NP, NPK, and NPKMg treatments were significantly lower than the Control treatment in all years, except in 2018, when the NPK and NPKMg treatments were significantly higher than the Control one (Table 7).

### 2.8. Incidence of Cherry Leaf Spot

Cherry leaf spot incidence was the lowest in 2022 for both cultivars across all four fertilizer treatments (below 15%), whereas the highest incidence was recorded in 2016 on cv. ‘Carmen’ in the control treatment (83.0%; Table 8). The ‘overall (treatments)’ data indicated that leaf spot incidence was significantly higher in cv. ‘Carmen’ compared to cv. ‘Vera’ in all years (*p* = 0.05; Table 8).

Significant differences in leaf spot incidence among the four fertilizer treatments were observed for both cultivars in 2016, 2017, 2018, and 2020 (Table 8). In these years, the NPKMg treatment consistently showed significantly lower incidence compared to the control for both cultivars. However, the overall values for the four fertilizer treatments (‘overall cultivars’) revealed no significant differences in any year (Table 8).

### 2.9. Correlation Among Parameters

**Figure 1:**
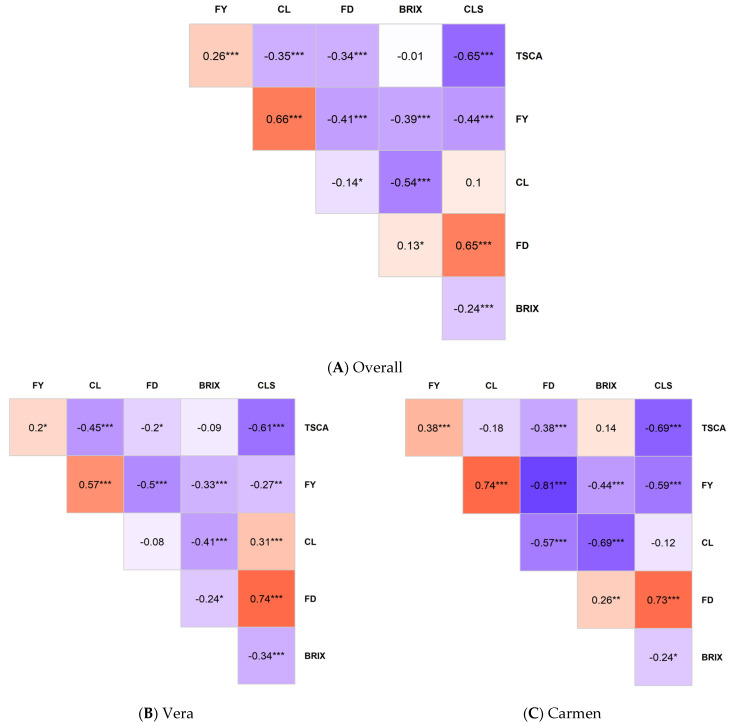

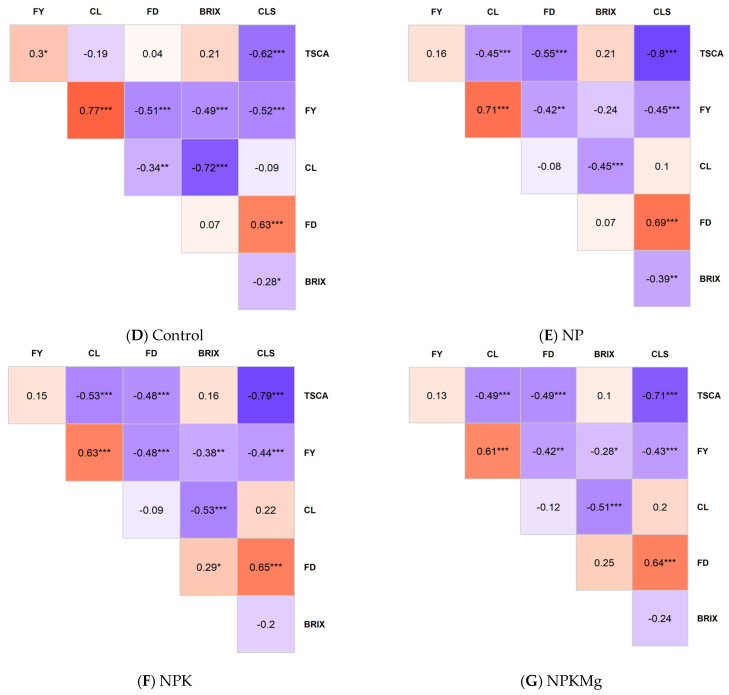
Pearson’s correlation coefficients (*r*) among six measured parameters for the overall dataset (**A**) for the two sweet cherry cultivars ‘Vera’ (**B**) and ‘Carmen’ (**C**), and for the four fertilizer treatments: Control (**D**), NP (**E**), NPK (**F**), and NPKMg (**G**) at Debrecen–Pallag, Hungary, over 2016–2022. The six measured parameters were trunk cross-sectional area (TCSA), fruit yield (FY), crop load (CL), fruit diameter (FD), water-soluble dry matter content (BRIX), and cherry leaf spot incidence (CLS). *, ** and *** indicate significance at *p* = 0.05, 0.01, and 0.001, respectively.

The highest Pearson correlation coefficient (*r* = 0.81) was observed between FD and FY in the cv. ‘Carmen’ data, including all fertilizer treatments (Figure 1C). In the overall dataset, only the correlation coefficients for BRIX vs. TCSA and CLS vs. CL were nonsignificant, while *r* values exceeded 0.5 for four parameter pairs: CLS vs. TCSA, CL vs. FY, BRIX vs. CL, and CLS vs. FD (Figure 1A).

For the individual cultivars, *r* values for cv. ‘Vera’ were nonsignificant only for BRIX vs. TCSA and FD vs. CL pairs, whereas for cv. ‘Carmen’, only BRIX vs. TCSA and CLS vs. CL pairs were nonsignificant (Figure 1B,C). For cv. ‘Vera’, *r* values exceeded 0.5 for three parameter pairs, namely CLS vs. TCSA, CL vs. FY, and CLS vs. FD, while for cv. ‘Carmen’, *r* values were above 0.5 for seven parameter pairs: CLS vs. TCSA, CL vs. FY, FD vs. FY, CLS vs. FY, FD vs. CL, BRIX vs. CL, and CLS vs. FD (Figure 1B,C).

For fertilizer treatments, the r values for the Control treatment were significant for CLS vs. TCSA, CL vs. FY, FD vs. FY, BRIX vs. FY, CLS vs. FY, FD vs. CL, BRIX vs. CL, CLS vs. FD, and CLS vs. BRIX; among these, *r* values exceeded 0.5 for six parameter pairs: CLS vs. TCSA, CL vs. FY, FD vs. FY, CLS vs. FY, BRIX vs. CL, and CLS vs. FD (Figure 1D). For the NP treatment, *r* values were nonsignificant for FY vs. TCSA, BRIX vs. TCSA, BRIX vs. FY, FD vs. CL, CLS vs. CL, and BRIX vs. FD. The *r* values exceeded 0.5 for three parameter pairs: FD vs. TCSA, CLS vs. TCSA, and CLS vs. FD (Figure 1E). For the NPK treatment, *r* values were nonsignificant for FY vs. TCSA, BRIX vs. TCSA, FD vs. CL, CLS vs. CL, and CLS vs. BRIX. The *r* values exceeded 0.5 for five parameter pairs: CL vs. TCSA, CLS vs. TCSA, CL vs. FY, BRIX vs. CL, and CLS vs. FD (Figure 1F).

For the NPKMg treatment, *r* values were nonsignificant for FY vs. TCSA, BRIX vs. TCSA, FD vs. CL, CLS vs. CL, BRIX vs. FD, and CLS vs. BRIX. The *r* values exceeded 0.5 for four parameter pairs: CLS vs. TCSA, CL vs. FY, BRIX vs. CL, and CLS vs. FD (Figure 1G).

### 2.10. Linear Regression Among Selected Parameters

The strongest overall correlations (across overall, cultivar, and fertilizer treatment comparisons) were CLS vs. TCSA, CL vs. FY, BRIX vs. CL, and CLS vs. FD; therefore, these relationships were further analyzed using regression analyses. Linear regression analysis revealed statistically significant relationships for all four variable pairs, with correlation coefficients ranging from *r* = 0.684 to 0.782 and *p*-values between 0.041 and 0.001 across the four fertilizer treatments. However, comparisons of slope parameters indicated no significant differences among the Control, NP, NPK, and NPKMg treatments, as confirmed by *t*-tests yielding *p*-values between 0.567 and 0.071. Similarly, slope parameters did not differ significantly between the two cultivars, with the exception of the FD vs. CLS relationship.

**Figure 2:**
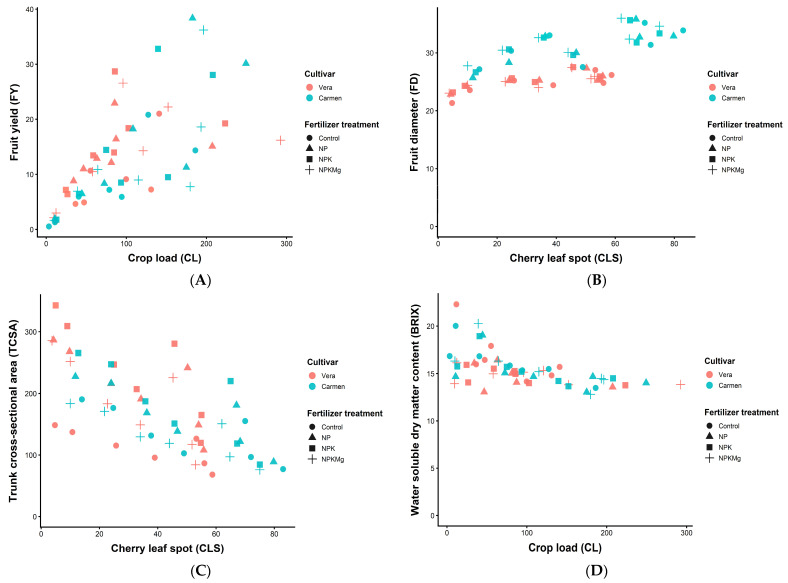
Relationships between four variable pairs: FY versus (vs). CL (**A**), FD vs. CLS (**B**), TCSA vs. CLS (**C**), and BRIX vs. CL (**D**) for the four fertilizer treatments (Control, NP, NPK, NPKMg) in an experimental sweet cherry orchard at Debrecen–Pallag, Hungary, over 2016–2022, on two sweet cherry cultivars, ‘Vera’ and ‘Carmen’ (n = 56; two cultivars × four fertilizer treatments × 7 years).

For the FY vs. CL relationship, increasing crop load was generally associated with higher fruit yield across both fertilizer treatments and cultivars (Figure 2A). In contrast, the FD vs. CLS relationship showed a clear separation between the two cultivars, where increasing cherry leaf spot incidence was accompanied by only a slight increase in fruit diameter under all fertilizer treatments (Figure 2B). In the TCSA vs. CLS relationship, most TCSA values increased proportionally with rising levels of cherry leaf spot incidence, regardless of fertilizer treatment or cultivar (Figure 2C). Finally, the BRIX vs. CL relationship indicated that increasing crop load had minimal influence on the water-soluble dry matter content of the fruits across all fertilizer treatments (Figure 2D).

### 2.11. Principal Component Analyses

The first five principal components (PCs) together explained 98.87% of the total variance and were therefore retained for further interpretation based on the cumulative variance criterion (Table 9), although only the first two PCs met the criterion (eigenvalues > 1; PC1 = 2.516, PC2 = 2.059), while PCs 3–5 had eigenvalues below 1.0 (0.730, 0.527, and 0.099, respectively). The root mean square residual (RMSR) was 0.05, indicating a satisfactory model fit. PC1 accounted for 41.94% of the total variance and was mainly associated with TCSA, FY, FD, and CLS (Table 9). PC2 explained 34.32% of the variance and was linked to TCSA, CL, BRIX, and CLS. PC3 contributed 12.17% of the variance and was primarily related to TCSA and BRIX. PC4 explained 8.78% of the variance and was associated with FY and FD, while PC5 accounted for 1.66% of the variance and was mainly related to FY and CLS (Table 9).

**Figure 3:**
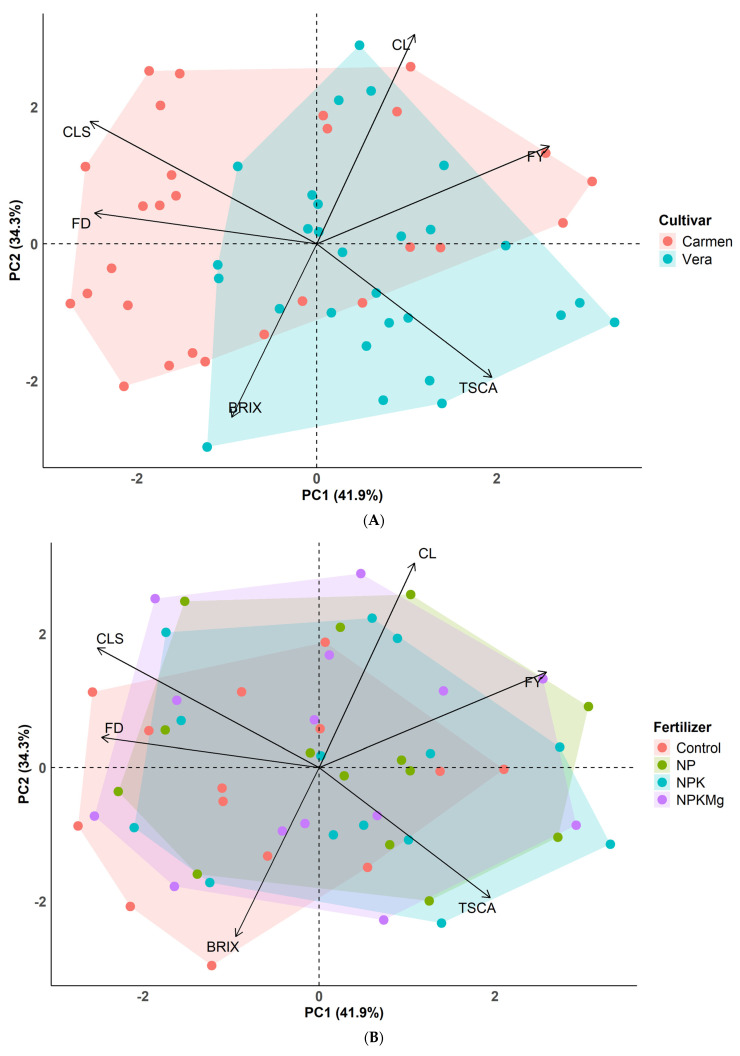
Biplot of principal component analysis (PCA) based on six measured variables: trunk cross-sectional area (TCSA), fruit yield (FY), crop load (CL), fruit diameter (FD), water-soluble dry matter content (BRIX), and cherry leaf spot incidence (CLS). The analysis includes four fertilizer treatments (Control, NP, NPK, NPKMg) and two sweet cherry cultivars (‘Vera’ and ‘Carmen’) evaluated in an experimental orchard at Debrecen–Pallag, Hungary, during 2015–2019. (**A**) 95% confidence intervals are shown separately for the two cultivars. (**B**) 95% confidence intervals are shown separately for the four fertilizer treatments.

In the biplot illustrating the two cultivars, both PC1 and PC2 axes contributed substantially to the observed variation (Figure 3A). Nevertheless, the distribution suggested a relatively stronger influence of PC1 for cv. ‘Vera’; whereas, for cv. ‘Carmen’, the effect of PC2 appeared more pronounced (Figure 3A). While in the biplot representing the four fertilization treatments, both PC1 and PC2 axes again accounted for a considerable proportion of the variability across treatments (Figure 3B). The treatments NP, NPK, and NPKMg were strongly associated with both axes, while the control treatment showed a greater alignment with the PC2 axis (Figure 3B).

The extended length and spatial orientation of the vectors corresponding to fruit diameter, cherry leaf spot incidence, crop load, and fruit yield indicated a predominant contribution to PC2. In contrast, trunk cross-sectional area and water-soluble dry matter content were more closely associated with PC1. Overall, these patterns point to strong interrelationships among tree performance parameters and the incidence of leaf spot disease (Figure 3A,B).

## 3. Discussion

In this 7-year study, the multivariate effects of four fertilizer treatments (Control, NP, NPK, NPKMg) were assessed on five tree parameters and the leaf spot incidence of two sweet cherry cultivars (‘Vera’ and ‘Carmen’). The effects of the four fertilizer treatments varied among the observed parameters depending on cultivars and years too.

### 3.1. Fruit Yield

Overall mean fruit yield was not significantly different between the two cultivars (12.9 and 13.0 kg tree^−1^ for cvs. ‘Vera’ and ‘Carmen’, respectively) over the 7-year period (Table 4), which contrasts with the results of Hrotkó et al. [37], who reported higher yield per tree for cv. ‘Vera’. This discrepancy likely reflects differences in soil conditions, training systems, and orchard management.

Another characteristic of our study is that fruit yield varied substantially between years, with strong reductions especially in 2020 due to partial spring frost damage (Table 4). Meteorological records support this interpretation, as minimum temperatures during flowering dropped to −7.5 °C in April 2020 (Table S1), indicating severe frost during sensitive reproductive stages. In contrast, warmer springs with moderate precipitation generally resulted in higher yields, confirming the strong dependence of sweet cherry productivity on spring thermal conditions and frost occurrence. The high susceptibility of sweet cherry to cold and frost stress during flowering and early fruit set is well documented, as low temperatures trigger physiological stress responses that can reduce fruit set and ultimately yield [38]. This underscores the vulnerability of sweet cherry to extreme weather conditions, consistent with studies identifying frost events as major limiting factors in cherry production [3,10]. In addition, climate-dependent variation in key agronomic traits, such as flowering time and fruit set, has been attributed to significant genotype × environment interactions in sweet cherry, reinforcing the strong environmental sensitivity of yield [39].

In our study, extreme yield variability among years (above 90%) significantly increased production risk. This was confirmed as mean yield during the seven-year period, which ranged between 1.5 and 28.7 kg tree^−1^ for cv. ‘Vera’ and between 0.5 and 38.5 kg tree^−1^ for cv. ‘Carmen’ (Table 4), showing great yield variability for both cultivars, with estimated coefficients of variation of 45.0% for ‘Vera’ and 48.7% for ‘Carmen’, indicating high yield instability among years for both cultivars.

Across the fertilization trial, NP, NPK, and NPKMg treatments generally increased yield compared to the control, confirming earlier findings that balanced nutrient management enhances productivity in sweet cherry [14,24]. However, the magnitude of the fertilization effect varied considerably by years.

Although no significant main effect of cultivar was detected across all years (Table 1), the significant cultivar × fertilizer interaction indicates that the response to fertilization was genotype-dependent. This suggests that cultivars differed in nutrient use efficiency and yield response patterns, resulting in similar overall means but contrasting responses under specific fertilization regimes. This highlights the importance of considering management-specific cultivar performance rather than relying on overall mean comparisons.

### 3.2. Trunk Cross-Sectional Area

Trunk cross-sectional area (TCSA) is widely recognized as a reliable indicator of vegetative vigor, strongly influenced by nutrient supply and environmental factors [20]. Comparing the cultivars, Hrotkó et al. [37] stated that both cultivars are characterized by moderate vigor. In our trial, cv. ‘Vera’ showed higher vigor, as trunk cross-sectional values were notably higher than those of cv. ‘Carmen’ (Table 2 and Table 3). This result contrasts with the findings of Bujdosó et al. [40], who reported higher vigor for cv. ‘Carmen’ in a study evaluating the effect of seven rootstocks.

Regarding fertilizer treatments, cv. ‘Vera’ showed significant differences in TCSA in each year, whereas cv. ‘Carmen’ responded only in 2017 (Table 2). Similar cultivar-specific differences in response to nitrogen fertilization have also been reported in sweet cherry [13,15]. These findings suggest that cv. ‘Vera’ is more sensitive to fertilization, while cv. ‘Carmen’ may be more influenced by external stress factors such as frost damage or crop load. Similar patterns of cultivar-dependent nutrient responses have been reported in previous cherry works [20,27,28].

Importantly, our ANOVA revealed a significant cultivar × year interaction for TCSA (Table 1), indicating that the relative vegetative vigor of the two cultivars varied among years. This interannual variation can be attributed to climatic conditions, including temperature, precipitation, and frost events, which differentially modulate growth responses. In our experimental years, low annual precipitation combined with elevated summer temperatures may have restricted vegetative growth in some treatments, whereas years with higher precipitation likely promoted trunk expansion (Table S1). In some years, favorable environmental conditions amplified the vigor advantage of cv. ‘Vera’, whereas in years with stress events such as frost or high crop load, differences between cultivars were reduced. Such genotype × environment × management interactions have also been reported in sweet cherry, emphasizing the importance of accounting for annual environmental variability when interpreting cultivar performance [13,20].

### 3.3. Crop Load

In our study, crop load showed large interannual variation between cultivars: in some years cv. ‘Vera’ exhibited higher values, whereas in other years cv. ‘Carmen’ performed better, while the overall 7-year effect was not significantly different between the two cultivars (Table 5). These results are partially in contrast with Hrotkó et al. [37], who reported no significant differences between the two cultivars, and also with Bujdosó et al. [40], who found higher productivity for cv. ‘Vera’ (0.36 kg cm^−2^) compared with cv. ‘Carmen’ (0.20 kg cm^−2^) in a long-term trial, highlighting the strong influence of environmental and management conditions on cultivar performance. These findings also demonstrate that crop load is highly sensitive to both environmental variability and nutrient management, consistent with previous studies showing that crop load is strongly influenced by nutrient availability and tree vigor [6,9,41].

In our trial, higher fertilizer inputs significantly increased crop load in several years, particularly in cv. ‘Vera’ (Table 5). These results are consistent with previous studies demonstrating that nutrient availability directly influences fruiting intensity, and that increased crop load may be associated with reduced fruit diameter and fruit quality due to assimilate competition within the canopy [6,9,41]. In this study, year-to-year variability in crop load was likely associated with meteorological conditions. Frost events during flowering in 2020 and 2021, combined with uneven precipitation during fruit set and early fruit development, likely contributed to reduced and unstable crop loads (Table S1).

The significant cultivar × fertilizer interaction detected by ANOVA (Table 1) indicates that the effect of fertilization on crop load was genotype-dependent, as the increase in fruiting intensity due to fertilization was not uniform across cultivars: cv. ‘Vera’ responded more strongly to NP, NPK, and NPKMg treatments in multiple years, whereas cv. ‘Carmen’ showed more variable responses depending on the season. Such genotype-specific nutrient responses are consistent with sweet cherry physiology, where the balance between vegetative growth and reproductive output is modulated by both cultivar genetics and external inputs [13,15]. Furthermore, high crop load may induce assimilate competition, potentially reducing fruit diameter and fruit quality [6,9]. The present findings underscore that both cultivar selection and fertilization strategy should be considered jointly in sweet cherry orchard management to achieve balanced crop load and stable production.

### 3.4. Fruit Diameter

Fruit diameter is widely recognized as a key quality trait in sweet cherry, as it directly determines market value [4,42], with a commonly accepted market threshold of around 28 mm. In this study, fruit diameter was strongly cultivar-dependent, as cv. ‘Carmen’ consistently reached or exceeded this standard, whereas cv. ‘Vera’ remained below this threshold throughout the trial (Table 6). These results are in accordance with previous findings [43,44,45,46], which reported that cv. ‘Vera’ typically produces fruits of 24–27 mm, while cv. ‘Carmen’ produces 30–32 mm under irrigated orchard conditions.

Fertilization generally improved fruit diameter, particularly under NP and NPK treatments, demonstrating the importance of balanced nutrient supply in promoting fruit growth [17]. Fruit diameter also appeared to be influenced by seasonal climatic conditions, particularly water availability during fruit enlargement. Higher precipitation in late spring and early summer, such as in 2016 and 2020, likely promoted cell expansion and fruit growth, whereas drier conditions, such as in 2021, may have limited fruit enlargement despite fertilization (Table S1). However, the significant cultivar × fertilizer interaction observed in ANOVA (Table 1) indicates that the magnitude of the fertilization effect depended on cultivar identity: cv. ‘Vera’ showed more consistent increases in fruit size under multiple fertilization regimes, whereas cv. ‘Carmen’ responded more variably depending on year and treatment. This suggests that genetic potential defines the baseline fruit diameter, while nutrient management can modulate, but not fully override, inherent cultivar limitations. Understanding these genotype-specific responses is therefore essential for designing fertilization strategies that optimize fruit diameter and achieve market standards.

### 3.5. Water-Soluble Dry Matter Content

Water-soluble dry matter content (BRIX) was highest in the control treatments, whereas fertilized trees generally showed lower values (Table 7). This inverse relationship between yield and soluble solids is consistent with the dilution effect reported in both sweet and sour cherry [28,47]. While fertilization improves yield and fruit diameter, excessive nutrient supply may reduce sugar concentration due to enhanced fruit growth and water accumulation [25].

In terms of cultivar characteristics, Hrotkó et al. [37] reported higher soluble solids for cv. ‘Carmen’ (12.33–14.09 Brix%) than for cv. ‘Vera’ (11.03–12.91 Brix%). In our trial, however, water-soluble dry matter content ranged from 13.58 to 22.30 Brix% for cv. ‘Vera’ and from 12.80 to 20.26 Brix% for cv. ‘Carmen’, indicating that, beyond genetic differences, crop load and fertilization strongly influenced fruit quality. The observed alternation in cultivar superiority among years further supports the strong influence of environmental variability on soluble solid accumulation. Seasonal temperature and precipitation patterns likely contributed substantially to these fluctuations (Table S1). Warmer and drier summers may have promoted sugar concentration through reduced fruit water content, whereas higher precipitation during fruit maturation may have diluted soluble solid accumulation.

The significant cultivar × fertilizer interaction (Table 1) indicates that the effect of nutrient supply on soluble solids content was genotype-dependent. Although fertilization generally reduced BRIX values, the magnitude of this reduction differed between cultivars and among years. The significant cultivar × year interaction further suggests that annual climatic conditions—such as temperature, radiation, and water availability—differentially influenced sugar accumulation. The fertilizer × year interaction indicates that fertilization effects were season-dependent, likely reflecting interannual differences in crop load and tree water status. Moreover, the significant cultivar × fertilizer × year interaction highlights the complex interplay among genetic background, nutrient management, and environmental conditions in determining fruit quality.

Recent genetic studies further indicate that sugar accumulation in sweet cherry is not solely determined by environmental conditions and orchard management but is also under substantial genetic control. Gracia et al. [48] identified quantitative trait loci (QTLs) associated with sugar and acid content on linkage groups 2 and 5, which co-localized with maturity date QTLs, highlighting the strong genetic regulation of fruit quality traits. These findings support the cultivar-dependent soluble solid content responses observed in the present study and emphasize that fruit soluble solids content results from complex genotype × environment × management interactions rather than from crop load or fertilization effects alone.

These findings are supported by previous studies showing that sugar accumulation in sweet cherry is strongly governed by source–sink relationships and environmental conditions during fruit development, particularly light and temperature [6,49]. Overall, optimizing fruit quality requires an integrated management approach that aligns nutrient supply with cultivar characteristics and seasonal variability.

### 3.6. Cherry Leaf Spot Incidence

This 7-year study showed clear cultivar-dependent differences in susceptibility to cherry leaf spot, as disease incidence was consistently higher in cv. ‘Carmen’ than in cv. ‘Vera’ across all seasons (Table 8). Such genotype-related variation is well documented in sweet and sour cherry pathosystems, where cultivar architecture, leaf morphology, phenology, cuticular traits, and biochemical defense capacity collectively influence host susceptibility to *Blumeriella jaapii* infection [33,50,51]. Previous studies have likewise reported marked differences in disease severity among cherry genotypes, highlighting cultivar choice as a key component of integrated disease management [33,52,53]. The stable separation observed between cultivars in the present study further confirms that host genetic background remained a primary determinant of disease expression.

Although treatment effects were not significant in the pooled cultivar means, significant differences among fertilizer regimes were detected in several individual years (2016, 2017, 2018, and 2020), when the NPKMg treatment repeatedly showed lower disease incidence than the non-fertilized control (Table 8). This indicates that balanced nutrient supply may not eliminate disease pressure but can enhance tree tolerance and suppress epidemic development under conducive environmental conditions. This interpretation is supported by recent evidence showing that optimized mineral nutrition improves physiological resilience and indirectly reduces susceptibility to foliar pathogens through improved canopy function and nutrient homeostasis [34,54].

The beneficial effect of balanced fertilization likely arises from the integrated physiological roles of macronutrients. Potassium enhances osmotic regulation, stomatal control, and the activation of defense-related enzymes, while calcium strengthens cell wall integrity, limiting pathogen penetration [55,56]. Magnesium, as a central component of chlorophyll, supports photosynthetic efficiency and carbohydrate availability for defense responses [57,58]. Collectively, these nutrients promote canopy physiological stability and stress tolerance, which may reduce disease progression under conducive environmental conditions [11,35].

By contrast, nitrogen excess is widely recognized as a risk factor for increased susceptibility to fungal diseases in perennial fruit crops. High nitrogen availability promotes excessive vegetative growth, increases canopy density, and modifies microclimatic conditions (e.g., humidity and leaf wetness duration), all of which favor infection by foliar pathogens [33,34,54,59]. In cherry, vigorous shoot growth induced by high nitrogen supply has been associated with increased leaf susceptibility and reduced structural resistance to infection [34,36]. However, autumn nitrogen application can enhance leaf litter decomposition, thereby reducing overwintering inoculum of *B. jaapii* and decreasing leaf spot incidence in the following spring [60,61,62]. These mechanisms may explain why treatments with unbalanced nutrient ratios did not consistently suppress disease incidence.

Annual variability in disease severity was pronounced, with low incidence observed in 2022 and severe epidemics recorded in 2016 (Table 8). These differences were closely associated with seasonal precipitation patterns. Wet conditions in 2016 (720 mm annual precipitation), particularly during late spring and summer, likely promoted repeated infection cycles and prolonged leaf wetness favorable for *B. jaapii* development, whereas drier conditions in 2021 (397 mm) and partly in 2022 may have limited epidemic development despite the presence of susceptible host tissue (Table S1). Such fluctuations reflect the strong influence of meteorological conditions on *B. jaapii* epidemiology, particularly rainfall patterns, temperature, and leaf wetness duration [33,63]. Environmental forcing often overrides management effects at the seasonal scale, as epidemic development strongly depends on primary inoculum availability and infection cycles [64]. Similar strong year effects have been reported in epidemiological studies of cherry leaf spot, where weather conditions explain a major proportion of disease variability [33].

An essential factor in this study is that the orchard was managed under an integrated fungicide program. Therefore, differences among nutrient treatments represent residual nutritional effects under controlled disease pressure. In commercial systems, where fungicide pressure or spray timing varies, nutrient-driven effects may be more pronounced [33,65]. These results highlight balanced fertilization as a complementary component of integrated disease management rather than a standalone control strategy.

Overall, the results indicate that cultivar susceptibility and environmental conditions were the primary drivers of cherry leaf spot incidence, while balanced NPKMg fertilization reduced disease severity in epidemic years. These results indicate that nutrient management modulates host–pathogen interactions and should be integrated into orchard strategies combining cultivar selection, canopy architecture, and chemical protection.

### 3.7. Intercorrelation and Multivariate Insight

The correlation structure among measured traits revealed a coherent physiological network linking vegetative growth, reproductive output, fruit quality, and disease incidence (Figure 1, Figure 2 and Figure 3; Table 9). Across datasets, strong positive associations were consistently observed between fruit yield (FY) and fruit diameter (FD), as well as between canopy traits (trunk cross-sectional area, TCSA; crop load, CL) and productivity parameters, indicating tightly coupled source–sink relationships in sweet cherry trees. These findings agree with previous studies identifying tree vigor and canopy development as key determinants of assimilate supply and fruit growth in high-density systems [13,40,49].

The exceptionally strong FD–FY correlation in cv. ‘Carmen’ (*r* = 0.81) indicates cultivar-specific strengthening of sink–source coordination, suggesting higher sensitivity of fruit diameter to assimilate availability in this genotype (Figure 1). Similar genotype-dependent variability in trait integration has been reported in sweet cherry, where genetic background modulates the balance between vegetative and reproductive performance [39,51]. In contrast, weak or nonsignificant correlations involving soluble solid content indicate partial decoupling of sugar concentration from structural growth traits under the present conditions. This supports evidence that fruit quality is influenced not only by sink strength but also by environmental and management factors such as canopy light distribution and crop load regulation [4,9,21].

Principal component analysis reduced trait complexity into five components explaining 98.87% of the total variance (Figure 3). PC1, associated with TCSA, FY, FD, and leaf spot incidence, represents an integrated axis of tree vigor, productivity, and disease expression. This aligns with studies showing that canopy structure and vegetative vigor jointly shape fruit production and microclimatic conditions favoring foliar disease development [22,23,33,34]. PC2, linked to TCSA, CL, BRIX, and leaf spot incidence, further highlights trade-offs among crop load, fruit quality, and disease pressure, indicating that assimilate allocation efficiency is central to yield–quality balance under variable nutrient regimes. Similar multivariate trade-offs have been reported in intensively managed cherry orchards [14,20,57].

Importantly, PCA loadings showed clustering of disease incidence with vegetative vigor and productivity traits, confirming that cherry leaf spot is embedded within the broader physiological system rather than acting as an isolated variable. This supports evidence that canopy density, leaf physiology, and nutrient-driven growth jointly regulate host susceptibility to *B. jaapii* [33,34,64].

Regression analysis reinforced these patterns, showing stable linear relationships between key trait pairs across treatments and cultivars (Figure 2). The absence of significant differences in slopes among treatments indicates that fertilization primarily affected trait magnitude rather than altering fundamental allometric relationships, suggesting a conservative scaling architecture in sweet cherry growth under nutrient variation [11,49].

Overall, the multivariate framework demonstrates that sweet cherry performance is governed by a tightly interconnected trait network in which vegetative growth, yield, fruit quality, and disease incidence form an integrated physiological system. Within this framework, cultivar identity acts as a key structuring factor, while nutrient management modulates interaction strength rather than network topology. Meteorological variability further influenced this integrated trait network by simultaneously affecting vegetative growth, fruit development, and disease pressure. Frost events, summer temperature patterns, and seasonal precipitation likely acted as higher-level environmental drivers, modulating the strength and stability of physiological relationships across years.

## 4. Materials and Methods

### 4.1. Location of the Trial, Planting Design, Orchard Management Practices, and Meteorological Observations

A long-term field experiment was conducted over a seven-year period (2016–2022) in an experimental sweet cherry orchard located at the Pallag Experimental Station of the University of Debrecen (Debrecen–Pallag, Hungary; 47°35′31.5″ N, 21°38′19.3″ E). The main physicochemical characteristics of the soil are presented in Table 10. The site is characterized by a light-textured, sandy soil with low humus content and slightly alkaline conditions (pH 7.5–7.6). Soil nitrogen levels were below the optimal range, whereas the concentrations of phosphorus, potassium, and magnesium in the topsoil were within the optimal range for sandy soils, based on the Agricultural Technical Guidelines [66].

The experimental orchard was established in the spring of 2012. The trees were grafted onto *Prunus mahaleb* rootstock and trained to a free spindle canopy, with a spacing of 2.5 × 5.0 m, resulting in a planting density of 800 trees per hectare. At the onset of the experimental period in 2016, the trees were four years old and had reached an average height of approximately 4.0 m. Two Hungarian-bred sweet cherry cultivars were evaluated: ‘Vera’ and ‘Carmen’. The fruit size of cv. ‘Carmen’ is large, and the trees exhibit moderate vegetative vigor. Cultivar ‘Vera’ is characterized by a semi-upright, moderately vigorous growth habit, with good productivity and medium-sized fruits [43,44].

Orchard management practices followed the European Integrated Fruit Production (IFP) guidelines [70]. The plantation was equipped with a drip irrigation system. Winter pruning was carried out annually in February.

Meteorological data, including daily mean and minimum temperatures as well as precipitation, were recorded from January 2016 to December 2022 using a Metos agrometeorological station installed at the experimental site.

### 4.2. Fertilizer Treatments

Four fertilizer treatments (NP, NPK, NKPMg, and control) were applied in an experimental sweet cherry orchard over a seven-year period (2016–2022) (Table 11).

The treatments involved four various active ingredients: nitrogen (N), phosphorus (P_2_O_5_), potassium (K_2_O), and magnesium (MgO). Given that both cultivars fall into the same category of moderate vegetative vigor, their nutrient demands may be considered equivalent [37]. The application rates were 60 kg N ha^−1^, 80 kg P ha^−1^, 100 kg K ha^−1^, and 30 kg MgO ha^−1^. Control trees received no fertilizer throughout the entire experimental period. Nitrogen was supplied using Genezis Pétisó mineral fertilizer (Genezis Ltd., Pétfürdő, Hungary), which contains 27% nitrogen. Phosphorus was applied in the form of Genezis Szuperfoszfát (Genezis Ltd., Pétfürdő, Hungary), containing 18% P_2_O_5_. Potassium was provided using Genezis Kálisó (Genezis Ltd., Pétfürdő, Hungary), with an active K_2_O content of 60%, while magnesium was supplied through Keserűsó Espo Top fertilizer, containing 16% MgO.

For each treatment and cultivar, seven trees were selected per plot, with four replications in total. Within each plot, the central five trees were used for data collection, while the first and seventh trees of each plot functioned as buffer plants and were omitted from all measurements. Nitrogen fertilizer was applied annually in spring (7 April, 22, 29, 20, 17, 19, 13 March from 2016 to 2022, respectively) to minimize winter nutrient leaching, while P, K, and Mg fertilizers were applied annually in autumn (16, 15, 14, 15, 18, 17 November from 2016 to 2021, respectively). All fertilizers were applied annually to the soil, 1 m from the trunk, on both sides of the tree. The applied fertilizers were gently incorporated into the upper soil layer to a shallow depth. In the control plots, the soil was similarly loosened to the same depth, but no fertilizers were applied.

### 4.3. Assessment of Five Tree Parameters

Five tree parameters were assessed: trunk cross-sectional area (TCSA), fruit yield (FY), crop load (CL), fruit diameter (FD) and water-soluble dry matter content (BRIX). For each cultivar, five trees per assessment plot were selected as replicates for the measurement of all parameters.

Trunk thickness was determined annually in November, after leaf fall, using a Vernier caliper at the midpoint between the graft union and the basal main branches. Trunk cross-sectional area was calculated from trunk diameter measurements and expressed in cm^2^. For TCSA, yearly differences were computed using 2016 as the reference year (set to 0), followed by the calculation of successive differences: Y2017–Y2016, Y2018–Y2017, Y2019–Y2018, Y2020–Y2019, Y2021–Y2020, and Y2022–Y2021.

Fruit picking was performed at the biological maturity stage of both cultivars. Harvest dates were 6, 10, 2, 12, 15, 21, and 15 June for cv. ‘Vera’, and 14, 15, 11, 14, 22, 24, and 24 June for cv. ‘Carmen’ from 2016 to 2022, respectively. Fruit yield (kg tree^−1^) was determined by weighing the total harvested fruit from each selected tree. Crop load was calculated by dividing fruit yield by the trunk cross-sectional area (TCSA) and expressed as kg cm^−2^. Fruit diameter (mm) was measured using a Vernier caliper on a sample of 20 fruits per tree, corresponding to 100 fruits per treatment. The water-soluble solid content (Brix %) was measured using a digital ATAGO refractometer (PAL-1, ATAGO Co., Ltd., Tokyo, Japan). Approximately 0.1–0.2 mL of juice was placed on the refractometer prism, which was calibrated with distilled water prior to measurements. Each sample was measured in four replicates, and the mean value was recorded. Measurements were performed at room temperature (20–22 °C), with the prism cleaned between samples.

### 4.4. Assessment of Cherry Leaf Spot Incidence

**Figure 4:**
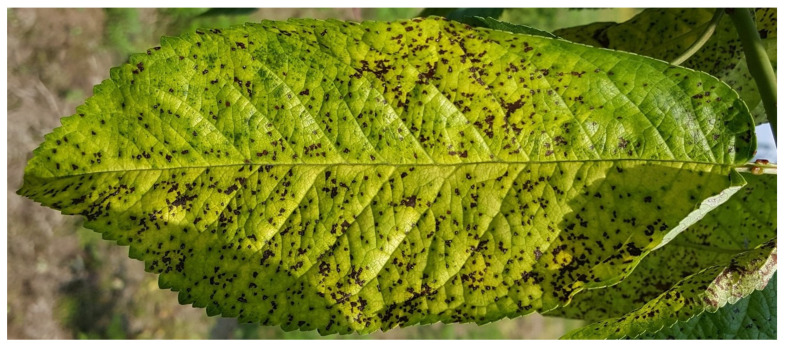
Representative symptoms of cherry leaf spot (*Blumeriella jaapii*) on a leaf of sweet cherry cultivar ‘Carmen’. Photo by I.J. Holb.

Cherry leaf spot [*Blumeriella jaapii* (Rehm) Arx, teleomorph; *Phloeosporella padi*, anamorph] was assessed on leaves for each fertilizer treatment, cultivar, and year. For each selected tree, 25 leaves per quadrant were examined at the end of September, resulting in 100 leaves per tree (4 × 25 leaves), and a total of 5 × 4 × 25 leaves per assessment plot. A leaf was considered diseased if it exhibited at least one visible leaf spot lesion (Figure 4). Cherry leaf spot incidence (CLS) was calculated as the average percentage of diseased leaves across the four quadrants of each tree. The same trees were used for cherry leaf spot assessments as for other measured tree parameters for both cultivars, with assessments conducted on 26, 29, 27, 25, 30, 28, and 30 September from 2016 to 2022.

### 4.5. Data Analyses

#### 4.5.1. Analysis of Variance (ANOVA)

For each year, fertilizer treatment, cultivar, and data from the six measured parameters (TCSA, FY, CL, FD, BRIX and CLS)) were averaged to produce a single value per tree. The resulting dataset was analyzed using analysis of variance (ANOVA) to evaluate the effects of year, fertilizer treatment, cultivar, and their interactions on all parameters. When F-tests were significant (*p* < 0.05), mean comparisons were conducted using the least significant difference (LSD) test at the 0.05 significance level. Percentage data for cherry leaf spot incidence were arcsine-transformed before analysis to satisfy the assumptions of normality. All statistical analyses were performed using Genstat Release 9.1 (Lawes Agricultural Trust, IACR, Rothamsted, UK).

#### 4.5.2. Correlation and Linear Regression Analyses

Relationships among the six measured parameters were evaluated using Pearson correlation coefficients (*r*), with significance assessed at *p* = 0.05. Correlations were calculated for the overall dataset, as well as separately for the two cultivars (‘Vera’ and ‘Carmen’) and for the four fertilizer treatments (Control, NP, NPK, and NPKMg). Pairs of parameters showing the strongest significant correlations (*r* > 0.5) were identified for the overall dataset, each cultivar, and each fertilizer treatment. These strongly correlated pairs were then plotted, and linear regression models were fitted for each fertilizer treatment. A *t*-test was applied to determine whether regression slopes differed significantly (*p* = 0.05) among the four fertilizer treatments or between cultivars. Statistical analyses were conducted using Genstat Release 9.1 (Lawes Agricultural Trust, IACR, Rothamsted, UK). Correlation matrices were visualized as heatmaps and simple linear regression analyses were graphically represented in R software 4.5.2 [71] using the ggplot2 3.5.1 [72], reshape2 1.4.4 [73], and dplyr 1.1.4 [74] packages.

#### 4.5.3. Principal Component Analysis

Principal component analysis (PCA) was conducted to examine multivariate relationships among the six measured parameters. All variables were standardized to have zero mean and unit variance before analysis. The PCA was performed using the correlation matrix, and components with eigenvalues greater than one were considered significant. The proportion of total variance explained by each principal component was calculated, and the first two components (PC1 and PC2) were used for visualization. PCA and biplot visualization were performed in R software 4.5.2 [71] using the ggplot2 3.5.1 [72], dplyr 1.1.4 [74], and ggrepel 0.9.6 [75] packages, while data handling was supported by the readxl 1.4.5 [76] and openxlsx 4.2.8 [77] packages.

## 5. Conclusions

This seven-year field study demonstrated that long-term N-P-K-Mg fertilization exerts a strong and multifaceted influence on both vegetative and generative performance of sweet cherry. Across cultivars, fertilization generally increased trunk cross-sectional area, yield, crop load, and fruit diameter compared with the control, confirming the central role of nutrient supply in orchard productivity. Leaf spot incidence showed strong interannual variability and was primarily driven by environmental conditions, with significant fertilization effects occurring only under high epidemic disease pressure. Disease responses were therefore highly conditional, reflecting interactions between epidemic severity and cultivar identity. Environmental factors exerted a dominant influence on both yield formation and disease development, frequently overriding fertilization effects. Overall, responses were cultivar-dependent, indicating consistent physiological differences between cvs. ‘Vera’ and ‘Carmen’.

Multivariate analyses revealed strong interrelationships among vegetative growth, crop load, fruit parameters, and cherry leaf spot, demonstrating that sweet cherry performance is governed by tightly connected trait networks rather than isolated responses. Yield and fruit diameter showed consistently strong positive associations, while disease incidence was indirectly linked to canopy vigor and fruit growth dynamics.

In conclusion, sustainable sweet cherry production requires integrated, cultivar-specific nutrient management strategies that consider multivariate trait interactions and pronounced environmental variability. Long-term system-level approaches are therefore essential to optimize tree performance and disease regulation under Central European production conditions.

## Figures and Tables

**Table 1:** Analysis of variance for the effects of years (2016–2022), fertilizer treatments (Control, NP, NPK, NPKMg) and cultivars (‘Vera’ and ‘Carmen’) on trunk cross-sectional area (TCSA), fruit yield (FY), crop load (CL), fruit diameter (FD), water-soluble solid content (BRIX), and cherry leaf spot incidence (CLS). df: degrees of freedom; MS: mean squares; *p*: the probability values associated with the F-tests.

		TCSA			FY			CL	
Source of Variation	df	MS	*p*	df	MS	*p*	df	MS	*p*
Cultivar (C)	1	61,874	<0.001	1	0.56	0.826	1	13,119	0.003
Fertilizer (F)	3	98,775	<0.001	3	2244	<0.001	3	126,200	<0.001
Year (Y)	6	80,601	<0.001	6	594	<0.001	6	12,120	0.003
C × F	3	1217	0.051	3	265	0.001	3	23,931	0.002
C × Y	6	19,108	0.003	6	28.1	0.067	6	2617	0.061
F × Y	18	2008	0.045	18	37.6	0.063	18	3558	0.051
C × F × Y	18	834	0.069	18	60.3	0.051	18	2535	0.060
Total	168			168			168		
		**FD**			**BRIX**			**CLS**	
	**df**	**MS**	***p***	**df**	**MS**	***p***	**df**	**MS**	***p***
Cultivar (C)	1	2317	<0.001	1	6.76	<0.001	1	11,032	<0.001
Fertilizer (F)	3	156	<0.001	3	76.7	<0.001	3	16,597	<0.001
Year (Y)	6	3.59	0.001	6	29.8	<0.001	6	223	0.035
C × F	3	19.9	<0.001	3	10.1	<0.001	3	184	0.051
C × Y	6	1.85	0.065	6	5.39	<0.001	6	7.54	0.427
F × Y	18	1.32	0.053	18	3.48	0.001	18	11.7	0.114
C × F × Y	18	1.47	0.055	18	3.31	0.001	18	3.50	0.979
Total	168			168			168		

**Table 2:** Trunk cross-sectional area (TCSA, cm^2^) of two sweet cherry cultivars (‘Vera’ and ‘Carmen’) in four fertilizer treatments (Control, NP, NPK, NPKMg) (Debrecen–Pallag, Hungary, 2016–2022). ns: nonsignificant. Differences among treatments were evaluated using LSD_0.05_ at the 5% significance level. Means followed by different letters indicate statistically significant differences at *p* = 0.05 according to the LSD test. The absence of letters denotes that no significant differences were detected among treatment means.

Treatments	2016	2017	2018	2019	2020	2021	2022	Overall (Year)
**Vera**								
Control	68.0 b	86.5 c	95.6 c	115.3 c	126.5 c	137.3 c	148.5 b	111.1 b
NP	108.0 a	148.8 a	191.0 a	215.9 ab	241.8 ab	267.9 ab	286.5 a	208.6 a
NPK	119.7 a	164.9 a	207.1 a	247.0 a	280.5 a	309.2 a	342.8 a	238.7 a
NPKMg	84.2 b	117.1 b	148.9 b	183.3 b	225.7 b	251.9 b	285.5 a	185.2 a
LSD_0.05_	17.6	22.0	32.2	38.1	46.2	48.1	66.2	39.6
**Carmen**								
Control	77.0 ab	97 b	102.7 c	131.4 b	155.1 bc	176.4 b	190.4 c	132.8 b
NP	89.0 a	122 a	138.4 ab	168.7 a	181.0 b	216.6 a	227.6 b	163.3 a
NPK	84.5 ab	119 a	151.1 a	187.3 a	220.2 a	247.3 a	265.5 a	182.1 a
NPKMg	76.0 b	97 b	118.9 bc	129.6 b	150.9 c	171.0 b	183.7 c	132.5 b
LSD_0.05_	12.7	13.2	26.2	30.2	28.9	33.8	37.1	26.8
**Overall (cultivars)**								
Control	72.5 b	91.5 b	99.2 c	123.4 b	140.8 c	156.8 c	169.5 c	122.0 c
NP	98.5 a	135.5 a	164.7 a	192.3 a	211.4 ab	242.3 ab	257.1 ab	186.0 ab
NPK	102.1 a	141.8 a	179.1 a	217.2 a	250.4 a	278.3 a	304.1 a	210.4 a
NPKMg	80.1 b	107.1 b	133.9 b	156.5 b	188.3 b	211.5 b	234.6 b	158.9 b
LSD_0.05_	14.8	19.4	28.9	33.4	39.9	41.8	52.0	33.5
**Overall (treatments)**								
**Vera.**	95.0 a	129.3 a	160.7 a	190.4 a	218.6 a	241.6	265.8 a	185.9 a
**Carmen**	81.6 b	108.6 b	127.8 b	154.3 b	176.8 b	202.8	216.8 b	152.7 b
**LSD_0.05_**	12.6	18.7	27.6	32.2	37.0	ns	47.2	31.2

**Table 3:** Annual increase in trunk cross-sectional area (TCSA, cm^2^) in two sweet cherry cultivars (‘Vera’ and ‘Carmen’) under four fertilizer treatments (Control, NP, NPK, NPKMg) in Debrecen–Pallag, Hungary, 2016–2022. Annual TCSA increases were calculated using 2016 as the reference year (0), with subsequent yearly changes expressed as Y2017–Y2016, Y2018–Y2017, Y2019–Y2018, Y2020–Y2019, Y2021–Y2020, and Y2022–Y2021. LSD_0.05_ values presented based on Table 2. Means followed by different letters indicate significant differences at *p* = 0.05 according to the LSD test.

Treatments	2016	2017	2018	2019	2020	2021	2022
**Vera**							
Control	0.0	18.4 c	9.2 c	19.6 b	11.2 c	10.8 b	11.3 a
NP	0.0	40.8 a	42.2 a	24.9 b	25.8 b	26.2 a	18.6 a
NPK	0.0	45.2 a	42.2 a	39.9 a	33.5 ab	28.7 a	33.6 b
NPKMg	0.0	32.8 b	31.9 b	34.3 a	42.4 a	26.3 a	33.5 b
LSD_0.05_	-	7.3	10.1	9.3	10.2	6.4	8.2
**Carmen**							
Control	0.0	19.6 b	6.1 c	28.8 a	23.6 b	21.3 b	14.0 ab
NP	0.0	33.0 a	16.4 b	30.3 a	12.3 c	35.6 a	11.0 b
NPK	0.0	34.2 a	32.4 a	36.2 a	32.9 a	27.2 b	18.2 a
NPKMg	0.0	21.1 b	21.8 b	10.7 b	21.3 b	20.1 b	12.8 ab
LSD_0.05_	-	6.9	6.0	8.7	7.9	8.0	7.1
**Overall (cultivars)**							
Control	0.0	19.0 c	7.6 c	24.2 b	17.4 b	16.0 c	12.6 b
NP	0.0	36.9 a	29.3 ab	27.6 b	19.1 b	30.9 a	14.8 b
NPK	0.0	39.7 a	37.3 a	38.1 a	33.2 a	27.9 a	25.9 a
NPKMg	0.0	27.0 b	26.8 b	22.5 b	31.8 a	23.2 b	23.1 a
LSD_0.05_	-	7.1	8.0	8.9	9.1	7.1	8.0
**Overall (treatments)**							
Vera	0.0	34.3 a	31.4 a	29.7	28.2	23.0	24.2 a
Carmen	0.0	27.0 b	19.2 b	26.5	22.5	26.0	14.0 b
LSD_0.05_	-	7.0	8.1	ns	ns	ns	7.5

**Table 4:** Total fruit yield (kg tree^−1^) of two sweet cherry cultivars (‘Vera’ and ‘Carmen’) in four fertilizer treatments (Control, NP, NPK, NPKMg) (Debrecen–Pallag, Hungary, 2016–2022). ns: nonsignificant. Differences among treatments were evaluated using LSD_0.05_ at the 5% significance level. Means followed by different letters indicate significant differences at *p* = 0.05 according to the LSD test. The absence of letters denotes that no significant differences were detected among means.

Treatments	2016	2017	2018	2019	2020	2021	2022	Overall (Year)
**Vera**								
Control	7.2 b	4.9 b	9.1 c	1.5 b	4.6 bc	10.7 a	21.0	8.4 b
NP	15.1 a	12.2 a	16.4 b	12.9 a	11.0 b	8.8 a	22.9	14.2 a
NPK	19.3 a	14.0 a	18.4 ab	13.4 a	6.4 ab	7.2 a	28.7	15.3 a
NPKMg	16.2 a	14.3 a	22.2 a	10.5 a	2.1 c	3.0 b	26.6	13.6 a
LSD_0.05_	7.4	6.1	3.9	3.9	3.7	3.6	ns	5.1
**Carmen**								
Control	5.9 c	7.2	14.4 d	1.3 b	0.5 b	6.0 c	20.8 b	8.4 b
NP	11.3 a	8.3	30.1 a	6.5 a	1.9 a	18.2 a	38.4 a	14.2 a
NPK	9.5 ab	8.5	28.1 b	6.5 a	1.8 ab	14.5 ab	32.8 a	15.3 a
NPKMg	7.8 bc	9.0	18.6 c	6.9 a	1.6 ab	10.9 b	36.2 a	13.6 a
LSD_0.05_	2.2	ns	1.8	2.5	1.4	4.2	7.4	3.3
**Overall (cultivars)**								
Control	6.6 b	6.0 b	11.7 b	1.4 b	2.6 b	8.3 b	20.9 b	8.2 b
NP	13.2 a	10.2 a	23.3 a	9.7 a	6.5 a	13.5 a	30.6 a	15.3 a
NPK	14.4 a	11.2 a	23.2 a	10.0 a	4.1 ab	10.8 ab	30.7 a	14.9 a
NPKMg	12.0 a	11.6 a	20.4 a	8.7 a	1.9 b	6.9 b	31.4 a	13.3 a
LSD_0.05_	4.9	3.7	5.2	3.4	3.5	4.8	7.9	4.6
**Overall (treatments)**								
Vera.	14.4 a	11.3 a	16.5 b	9.6 a	6.0 a	7.4 b	24.8 b	12.9
Carmen	8.6 b	8.3 b	22.8 a	5.3 b	1.5 b	12.4 a	32.0 a	13.0
LSD_0.05_	3.4	2.8	4.5	3.1	2.1	3.2	5.7	ns

**Table 5:** Crop load (g cm^−2^) of two sweet cherry cultivars (‘Vera’ and ‘Carmen’) in four fertilizer treatments (Control, NP, NPK, NPKMg) (Debrecen–Pallag, Hungary, 2016–2022). ns: nonsignificant. Differences among treatments were evaluated using LSD_0.05_ at the 5% significance level. Means followed by different letters indicate statistically significant differences at *p* = 0.05 according to the LSD test. The absence of letters denotes that no significant differences were detected among treatment means.

Treatments	2016	2017	2018	2019	2020	2021	2022	Overall (Year)
**Vera**								
Control	131.0 c	47.3 b	99.8 b	11.8 b	36.6 ab	55.2 a	141.1 a	74.7 b
NP	207.3 b	81.3 ab	87.1 b	63.2 a	46.5 a	34.1 ab	85.4 b	86.4 ab
NPK	223.3 ab	84.7 ab	102.7 b	58.7 a	26.6 b	24.5 bc	85.7 b	86.6 ab
NPKMg	292.5 a	120.9 a	152.1 a	57.8 a	9.5 c	12.3 c	95.9 ab	105.9 a
LSD_0.05_	72.4	42.8	37.5	22.6	16.0	21.8	48.3	31.0
**Carmen**								
Control	94.4 b	78.8 b	186.2 b	10.9 b	3.4 b	40.5 c	127.4 b	77.4 b
NP	174.9 a	72.5 b	249.3 a	44.5 a	10.7 a	108.3 a	182.6 a	120.4 a
NPK	152.0 a	93.4 ba	207.9 ab	40.7 a	12.8 a	74.9 b	139.6 b	103.0 ab
NPKMg	179.7 a	115.0 a	193.3 ab	39.1 a	9.9 a	64.4 bc	196.4 a	114.0 a
LSD_0.05_	44.6	26.3	62.8	14.4	5.8	24.8	19.8	28.4
**Overall (cultivars)**								
Control	112.7 b	63.0 c	143.0	11.3 b	20.0 ab	47.9 ab	134.2	76.0 b
NP	191.1 a	76.9 bc	168.2	53.8 a	28.6 a	71.2 a	134.0	103.4 ab
NPK	187.7 a	89.0 b	155.3	49.7 a	19.7 ab	49.7 ab	112.6	94.8 ab
NPKMg	236.1 a	118.0 a	172.7	48.5 a	9.7 b	38.3 b	146.1	109.9 a
LSD_0.05_	53.7	23.9	ns	14.6	15.7	32.0	ns	32.9
**Overall (treatments)**								
Vera	213.5 a	83.5	110.4 b	47.9	29.8 a	31.5 b	102.0 b	88.4
Carmen	150.2 b	89.9	209.2 a	33.8	9.2 b	72.0 a	161.5 a	103.7
LSD_0.05_	43.3	ns	28.4	ns	9.0	18.1	24.8	ns

**Table 6:** Fruit diameter (mm) of two sweet cherry cultivars (‘Vera’ and ‘Carmen’) in four fertilizer treatments (Control, NP, NPK, NPKMg) (Debrecen–Pallag, Hungary, 2016–2022). ns: nonsignificant. Differences among treatments were evaluated using LSD_0.05_ at the 5% significance level. Means followed by different letters indicate statistically significant differences at *p* = 0.05 according to the LSD test. The absence of letters denotes that no significant differences were detected among treatment means.

Treatments	2016	2017	2018	2019	2020	2021	2022	Overall (Year)
**Vera**								
Control	26.2	24.8	24.4 b	25.2 b	27.0 b	23.5 b	21.3 b	24.6 b
NP	26.0	25.2	25.2 a	25.2 b	27.3 a	24.2 a	22.8 a	25.2 ab
NPK	25.9	25.6	25.0 a	25.6 a	27.5 a	24.3 a	23.2 a	25.3 a
NPKMg	25.9	25.5	24.0 b	25.0 b	27.5 a	24.4 a	23.0 a	25.1 ab
LSD_0.05_	ns	ns	0.58	0.35	0.29	0.52	1.26	0.61
**Carmen**								
Control	33.9 b	31.4 c	27.5 b	33.0	35.2	30.3 a	27.2	31.2 b
NP	32.9 c	32.9 a	30.0 a	32.9	35.8	28.3 b	25.7	31.2 b
NPK	33.2 c	31.8 bc	29.6 a	32.7	35.6	30.6 a	26.6	31.5 ab
NPKMg	34.6 a	32.5 ab	30.1 a	32.6	36.0	30.4 a	27.8	32.0 a
LSD_0.05_	0.62	0.69	0.57	ns	ns	0.61	ns	0.60
**Overall (cultivars)**								
Control	30.0	28.1	26.0	29.1	31.1	26.9	24.2	27.9
NP	29.4	29.0	27.6	29.0	31.6	26.3	24.3	28.2
NPK	29.6	28.7	27.3	29.1	31.6	27.4	24.9	28.4
NPKMg	30.3	29.0	27.0	28.8	31.7	27.4	25.4	28.5
LSD_0.05_	ns	ns	ns	ns	ns	ns	ns	ns
**Overall (treatments)**								
Vera.	26.0 b	25.3 b	24.7 b	25.3 b	27.3 b	24.1 b	22.6 b	25.0 b
Carmen	33.7 a	32.1 a	29.3 a	32.8 a	35.7 a	29.9 a	26.8 a	31.5 a
LSD_0.05_	0.41	0.50	0.64	0.23	0.36	0.56	0.95	0.51

**Table 7:** Water-soluble dry matter content (Brix%) of two sweet cherry cultivars (‘Vera’ and ‘Carmen’) in four fertilizer treatments (Control, NP, NPK, NPKMg) (Debrecen–Pallag, Hungary, 2016–2022). ns: nonsignificant. Differences among treatments were evaluated using LSD_0.05_ at the 5% significance level. Means followed by different letters indicate statistically significant differences at *p* = 0.05 according to the LSD test. The absence of letters denotes that no significant differences were detected among treatment means.

Treatments	2016	2017	2018	2019	2020	2021	2022	Overall (Year)
**Vera**								
Control	14.80 a	16.44 a	14.16 a	22.30 a	15.98 a	17.90 a	15.70 a	16.8 a
NP	13.58 c	15.04 b	14.08 a	16.42 b	13.06 d	16.10 b	15.04 c	14.8 b
NPK	13.76 b	15.26 b	14.00 a	15.52 c	14.06 b	15.92 c	14.98 c	14.8 b
NPKMg	13.84 b	15.32 b	13.82 b	14.98 d	13.94 c	16.14 b	15.14 b	14.7 b
LSD_0.05_	0.17	0.48	0.16	0.44	0.10	0.07	0.11	0.18
**Carmen**								
Control	15.34 a	15.84 a	13.48 c	20.02 b	16.84 a	16.83 a	15.48 a	15.34 a
NP	13.04 c	15.08 c	14.02 b	19.04 c	14.68 d	14.68 d	14.68 b	13.04 c
NPK	13.66 b	15.20 b	14.50 a	18.94 c	15.76 c	15.70 c	14.22 c	13.66 b
NPKMg	12.80 d	15.20 b	14.48 a	20.26 a	16.32 b	16.33 b	14.36 c	12.80 d
LSD_0.05_	0.15	0.12	0.14	0.17	0.16	0.14	0.13	0.14
**Overall (cultivars)**								
Control	15.1 a	16.1 a	13.8 b	21.2 a	16.4 a	17.4 a	15.6 a	16.5 a
NP	13.3 c	15.1 b	14.1 ab	17.7 b	13.9 c	15.4 c	14.9 b	14.9 b
NPK	13.7 b	15.2 b	14.3 a	17.2 b	14.9 b	15.8 bc	14.6 b	15.1 b
NPKMg	13.3 c	15.3 b	14.2 a	17.6 b	15.1 b	16.2 b	14.8 b	15.2 b
LSD_0.05_	0.36	0.26	0.31	1.98	0.95	0.49	0.34	0.69
**Overall (treatments)**								
Vera.	14.0	15.5	14.0	17.3 b	14.3 b	16.5 a	15.2 a	15.3
Carmen	13.7	15.3	14.1	19.6 a	15.9 a	15.9 b	14.7 b	15.6
LSD_0.05_	ns	ns	ns	1.59	0.71	0.58	0.30	ns

**Table 8:** Cherry leaf spot incidence (%) of two sweet cherry cultivars (‘Vera’ and ‘Carmen’) in four fertilizer treatments (Control, NP, NPK, NPKMg) at the end of September (Debrecen–Pallag, Hungary, 2016–2022). ns: nonsignificant. Differences among treatments were evaluated using LSD_0.05_ at the 5% significance level. Means followed by different letters indicate statistically significant differences at *p* = 0.05 according to the LSD test. The absence of letters denotes that no significant differences were detected among treatment means.

Treatments	2016	2017	2018	2019	2020	2021	2022	Overall (Year)
**Vera**								
Control	58.8 a	56.0 a	39.0 a	25.8	53.3 a	10.8	4.8	35.5 a
NP	55.8 ab	54.0 ab	34.3 b	24.0	50.3 a	9.8	4.3	33.2 ab
NPK	54.8 ab	55.0 ab	32.8 b	25.0	45.8 b	9.0	5.0	32.5 ab
NPKMg	53.0 b	51.8 b	34.0 b	22.8	45.3 b	10.0	3.8	31.5 b
LSD_0.05_	5.48	3.79	4.11	ns	4.05	ns	ns	3.89
**Carmen**								
Control	83.0 a	72.0 a	49.0 a	37.8	70.0 a	24.8	14.0	83.0
NP	79.8 a	68.3 ab	46.8 ab	36.3	67.0 ab	24.0	11.8	79.8
NPK	75.0 b	67.3 ab	45.8 ab	35.8	65.0 ab	24.0	12.8	75.0
NPKMg	75.0 b	64.8 b	44.0 b	34.0	62.0 b	21.8	10.0	75.0
LSD_0.05_	4.51	5.84	4.20	ns	5.45	ns	ns	4.76
**Overall (cultivars)**								
Control	70.9	64.0	44.0	31.8	61.6	17.8	9.4	42.8
NP	67.8	61.1	40.5	30.1	58.6	16.9	8.0	40.4
NPK	64.9	61.1	39.3	30.4	55.4	16.5	8.9	39.5
NPKMg	64.0	58.3	39.0	28.4	53.6	15.9	6.9	38.0
LSD_0.05_	ns	ns	ns	ns	ns	ns	ns	ns
**Overall (treatments)**								
Vera.	55.6 b	54.2 b	35.0 b	24.4 b	48.6 b	9.9 b	4.4 b	33.2 b
Carmen	78.2 a	68.1 a	46.4 a	35.9 a	66.0 a	23.6 a	12.1 a	47.2 a
LSD_0.05_	2.97	2.61	2.35	1.82	3.01	1.93	1.67	2.43

**Table 9:** Eigenvalues, proportions of explained variance, and eigenvectors obtained from principal component analysis (PCA) of six measured variables under four fertilizer treatments (Control, NP, NPK, NPKMg) in two sweet cherry cultivars (‘Vera’ and ‘Carmen’) are presented. The study was conducted in an experimental orchard in Debrecen–Pallag, Hungary, over the period 2016–2022. Statistically significant values (*p* < 0.05) are indicated in bold.

Items	PC1	PC2	PC3	PC4	PC5
*Eigenvalue*	2.516	2.059	0.730	0.527	0.099
Proportion of variance (%)	41.94	34.32	12.17	8.78	1.66
Cumulative variance (%)	41.94	76.26	88.43	97.21	98.87
*Eigenvectors*					
Trunk cross-sectional area—TCSA	**0.389**	**−0.389**	**0.599**	−0.216	−0.082
Fruit yield—FY	**0.517**	0.286	−0.094	**−0.467**	**−0.557**
Crop load—CL	0.217	**0.612**	−0.283	−0.168	0.345
Fruit diameter—FD	**−0.494**	0.089	0.278	**−0.759**	0.268
Water-soluble dry matter content—BRIX	−0.189	**−0.506**	**−0.654**	−0.329	−0.249
Cherry leaf spot incidence—CLS	**−0.504**	**0.358**	0.218	0.151	**−0.655**

**Table 10:** Soil characteristics (seven parameters) of the experimental site in Debrecen–Pallag, Hungary in 2016, with reference optimal ranges for sandy soils according to the Agricultural Technical Guidelines [65].

Soil Parameters	0–20 cm	20–40 cm	40–60 cm	Optimal Value
Humus content (%)	1.6	1.5	1.2	1.2–2.0
pH	7.6	7.6	7.5	5.7–7.6
NO_3_ + NO_2_^−^–N (mg kg^−1^)	3.02	5.10	4.36	8.0–10.0
AL-P_2_O_5_ (mg kg^−1^)	146	95	83	80
AL-K_2_O (mg kg^−1^)	351	330	301	100–120
AL-Mg (mg kg^−1^)	108	97	145	60
CaCO_3_ (m/m) %	0.33	0.35	0.21	<3%

In the soil samples, the AL-extractable concentrations of P, K, Ca, and Mg were determined following the method of Egnér et al. [67]. The NO_3_^−^ + NO_2_^−^–N content was measured according to the Skalar method [68]. The CaCO_3_ content was determined using the procedure described by Filep [69], applying a Scheibler-type calcimeter.

**Table 11:** Fertilization treatments and application rates of N, P_2_O_5_, K_2_O, and MgO (nitrogen—N, phosphorus—P_2_O_5_, potassium—K_2_O, and magnesium—MgO) in the experimental sweet cherry orchard at Debrecen–Pallag, Hungary (2016–2022).

Treatment/Active Ingredients	N (kg ha^−1^)	P_2_O_5_ (kg ha^−1^)	K_2_O (kg ha^−1^)	MgO (kg ha^−1^)
Control	0	0	0	0
NP	60	80	0	0
NPK	60	80	100	0
NPKMg	60	80	100	30

## Data Availability

Data will be provided for other scientists upon reasonable request.

## Supplementary Materials

The following supporting information can be downloaded at https://www.mdpi.com/article/10.3390/plants15101499/s1, Table S1. Monthly mean and minimum temperatures (°C) and total monthly precipitation (mm) in Debrecen-Pallag, Hungary, from 2016 to 2022.
