# Supplementary material for: Multivariate Analysis on Seven-Year Effects of Balanced N-P-K-Mg Fertilization on Productivity and Leaf Spot Incidence in Two Sweet Cherry Cultivars
Source: Plants (Basel). 2026 May 14;15(10):1499. doi: 10.3390/plants15101499 (PMC13210966; doi:10.3390/plants15101499)
Supplement: Supplementary file 1 [file plants-15-01499-s001.zip › plants-4303512-supplementary.pdf]

plants

Supplementary material:

Table S1. Monthly mean and minimum temperatures (°C) and total monthly precipitation (mm) in Debrecen-Pallag, Hungary, from 2016 to 2022.

	January	February	March	April	May	June	July	August	September	October	November	December	Sum
Mean temperature (°C)													
2016	-1.7	6.1	7.0	13.3	16.5	21.1	22.3	20.8	17.6	9.7	4.6	-1.8	-
2017	-6.0	2.1	9.2	10.7	17.2	22.2	22.3	23.2	16.4	10.8	5.7	2.9	-
2018	2.6	0.4	3.6	16.5	20.2	21.3	22.9	24.1	17.6	12.7	6.9	0.7	-
2019	-1.3	3.4	8.7	13.3	14.7	23.7	21.9	23.5	17.0	12.3	9.8	6.4	-
2020	-1.3	4.6	7.0	11.5	14.8	20.5	21.8	23.2	17.9	12.3	5.2	4.5	-
2021	1.4	2.3	5.3	9.0	15.1	22.8	25.0	21.2	16.4	9.5	5.4	1.3	-
2022	-0.2	4.1	5.2	9.7	17.9	23.1	23.9	23.8	15.8	12.2	6.8	2.6	-
Minimum temperature (°C)													
2016	-15.7	-4.1	-6.2	-1.0	3.0	7.1	9.6	7.9	3.4	-0.8	-5.1	-9.5	-
2017	-17.9	-5.0	-2.6	-0.7	3.5	9.6	9.1	7.7	2.0	-3.4	-5.1	-2.6	-
2018	-8.0	-13.4	-17	4.2	10.5	7.1	7.9	11.0	2.0	-1.3	-10.8	-12.1	-
2019	-13.4	-6.1	-5.6	1.2	5.2	11.4	8.3	9.2	1.1	-2.4	-3.6	-3.2	-
2020	-9.0	-7.9	-4.1	-7.5	0.8	6.6	9.7	10.2	6.5	2.6	-5.3	-6.4	-
2021	-11.7	-12.8	-7.9	-4.6	1.6	7.8	14.1	8.3	3.1	-3.4	-6.1	-8.7	-
2022	-11.4	-5.3	-9.0	-2.6	3.7	8.9	7.5	13.7	3.6	-1.8	-1.6	-7.3	-
Precipitation (mm)													
2016	63	91	48	15	44	118	78	49	62	89	56	6	720
2017	21	40	28	51	27	64	69	61	78	38	57	102	636
2018	27	58	69	25	49	65	36	25	17	13	39	44	466
2019	24	7	8	47	69	31	56	18	22	16	81	49	429
2020	24	53	39	17	33	147	101	29	52	73	15	28	611
2021	37	39	21	45	63	8	52	31	18	13	30	39	397
2022	8	13	10	49	39	13	38	10	153	8	49	92	482
